# Polyglycerol Adipate-Grafted Polycaprolactone Nanoparticles as Carriers for the Antimicrobial Compound Usnic Acid

**DOI:** 10.3390/ijms232214339

**Published:** 2022-11-18

**Authors:** Vincenzo Taresco, Isotta Tulini, Iolanda Francolini, Antonella Piozzi

**Affiliations:** 1 Department of Chemistry, The University of Nottingham, Nottingham NG7 2RD, UK; 2 Department of Chemistry, Sapienza University of Rome, 00185 Rome, Italy

**Keywords:** nanoparticles, polyglycerol adipate, polycaprolactone, usnic acid, microbial infections, drug release

## Abstract

Nanoparticle (NP) drug delivery systems are known to potentially enhance the efficacy of therapeutic agents. As for antimicrobial drugs, therapeutic solutions against drug-resistant microbes are urgently needed due to the worldwide antimicrobial resistance issue. Usnic acid is a widely investigated antimicrobial agent suffering from poor water solubility. In this study, polymer nanoparticles based on polyglycerol adipate (PGA) grafted with polycaprolactone (PCL) were developed as carriers for usnic acid. We demonstrated the potential of the developed systems in ensuring prolonged bactericidal activity against a model bacterial species, *Staphylococcus epidermidis*. The macromolecular architecture changes produced by PCL grafted from PGA significantly influenced the drug release profile and mechanism. Specifically, by varying the length of PCL arms linked to the PGA backbone, it was possible to tune the drug release from a burst anomalous drug release (high PCL chain length) to a slow diffusion-controlled release (low PCL chain length). The developed nanosystems showed a prolonged antimicrobial activity (up to at least 7 days) which could be used in preventing/treating infections occurring at different body sites, including medical device-related infection and mucosal/skin surface, where Gram-positive bacteria are commonly involved.

## 1. Introduction

In the last decade, controlled drug release systems have undergone some remarkable developments. Today, the research in the pharmaceutical field is aimed not only at synthesizing novel biologically active molecules but also at developing new drug administering systems able to control drug release or to target the drug to a specific body site. Nanocarriers using synthetic or natural polymers are among the most studied, thanks to the ability of some polymers to self-assemble into controlled conditions to give different types of nanostructures [1,2,3,4].

Commonly used synthetic polymers for controlled drug release are polyethers such as polyethyleneglycol (PEG), and aliphatic polyesters, such as polycaprolatone (PCL), polylactide (PLA), polyglycolide (PGL) and their copolymers [5]. These polymers have been FDA-approved for medical use [6].

The choice of the polymer type (polyether or polyester) to be used as a carrier in a controlled drug release system depends also on the hydrophobicity/hydrophilicity features of the drugs to be carried [7,8].

Polyesters can be successfully employed for the release of drugs with poor solubility in water since they are also mainly hydrophobic. However, this feature together with the good crystallinity possessed by these polymeric materials may negatively affect drug loading as well as polymer biodegradation [9,10,11].

The degree of crystallinity as well as the amphiphilic balancing can be altered by copolymerizing these aliphatic polyesters with PEG as a hydrophilic polymeric block. However, despite its exceptional properties, among others, biocompatibility and solubility in both water and organic solvents, PEG suffers a series of critical drawbacks. A linear PEG bears a limited number of exploitable functionalities: PEG has been reported to trigger different levels of hypersensitivity or toxicity (related to the chain length) and it is produced from oil-based building blocks [12].

In this context, the synthesis of copolymers containing the semicrystalline PCL polyesters chains grafted to an amorphous polymer could be a solution. In fact, to minimize the physical restriction related to the high crystalline regions of PCL, poly(glycerol adipate) (PGA) has been chosen as an amorphous grafting structure. Therefore, the present work reports the preparation and characterization of biodegradable nanoparticle systems based on polycaprolactone-grafted polyglycerol adipate (PGA-g-PCL) to be used for the delivery of the hydrophobic model drug, usnic acid. 

Enzymatically synthesized PGA has shown a variety of key desirable properties required for drug-delivery applications [13]. PGA and its derivatives can self-assemble into nanoparticles (NPs) and interact at different levels with small active molecules [14,15]. In addition, PGA is a functionalize-able, biocompatible and biodegradable polymer [16,17]. The grafting of multifunctional moieties along the side chain of PGA can (a) tune the interaction with small- and macromolecules; (b) facilitate further anchoring with other bioactive molecules; (c) modulate the amphiphilicity; and (d) tune the enzymatic degradation tendency of the final PGA-based system [14,15,18]. In addition, the adoption of PGA-based polymers can be an opportunity to exploit a sustainable synthetic route using a natural catalyst and very mild reaction conditions. For example, glycerol global production, as a side product of the biodiesel process, has been predicted to exceed 4000 million liters by 2026 [19].

The (+)-Usnic acid (2,6-diacetyl-7,9-dihydroxy-8,9b-dimethyl-1,3(2H,9bH)-dibenzofurandione, UA) is a secondary metabolite of several lichens extensively studied for its broad variety of biological features [20,21]. The most interesting property of UA is its antimicrobial activity against bacteria growing either in planktonic or in biofilm mode [22,23]. Biofilm-based infections are a serious human threat due to their high antibiotic tolerance with respect to planktonic cells [24,25].

The potency of UA against sessile-growing bacteria makes it attractive for the development of efficacious antibacterial formulations, also considering the worldwide antimicrobial resistance issue. The inclusion of UA in nanocarriers is considered a strategy to overcome some limitations of UA, especially its poor aqueous solubility (less than 10 mg/100 mL at 25 °C) and dose-dependent hepatotoxicity [20]. Several research groups are working on this topic [26,27,28,29,30], including our group, who developed different UA-loaded nanocarriers based on cationic polymers [1], carboxylated PLA [31], magnetic nanoparticles [32] and liposomes [33] in order to control UA pharmacokinetics and biodistribution while reducing its side effects.

Within a framework of broadening the platform of polymer nanocarriers for usnic acid, in the present work, a copolymer polycaprolactone-grafted polyglycerol adipate was synthesized by introducing the monomer ε-caprolactone (CL) into the polyglycerol adipate (PGA) side chain followed by its ring opening polymerization (ROP). To verify the influence of different side chain lengths on the copolymer physical properties and drug release capacity of the systems, PGAs grafted with three different PCL theoretical side chain lengths (10, 25 and 50 repeating units) were prepared. To compare the physical features of the PGA-g-PCLs with those of the respective homopolymers, PCL homopolymers having 10, 25 and 50 repeating units were also synthesized by using ethanol as the ROP initiator. All of the obtained polymers were loaded with usnic acid and characterized by thermal (differential scanning calorimetry, DSC) and spectroscopic analysis (^1^H-NMR and FTIR) as well as in terms of antimicrobial features.

## 2. Results and Discussion

Polyglycerol adipate was obtained by the polycondensation of glycerol and adipic acid by using immobilized lipase as a chemo- and regioselective catalyst. The polymerization conditions were set to have a mainly linear polymer, preserving the secondary hydroxyl groups of glycerol along its backbone [34]. The key feature of this polymer is that the available hydroxyl moieties in the side chain permit its functionalization via simple chemistry [35,36].

PGA is, therefore, a functionalize-able, biodegradable and biocompatible polymer, ideal for drug delivery applications [13,16,17]. Moreover, PGA shows the ability to self-assemble into NPs in aqueous media thanks to its amphiphilic characteristics, without the use of surfactants [37].

PGA was modified in a variety of ways [13], mainly by post-polymerization-functionalization, for the introduction of acyl groups of different lengths [38], arms [39] and even drugs [40,41].

In the present study, the PGA OH moiety was used as macroinitiator for the ROP of ε-caprolactone monomer, with the aim of altering the physical properties of the polymer and tuning its interaction with drugs, as well as its self-assembling behaviors and degradability tendency [18]. The synthesis of the PGA-g-PCLs was carried out following the procedure previously detailed, developed and well-established in the literature by the Kressler group [35,42,43]. These consisted of the addition of CL to a solution of PGA in THF at different CL/OH-PGA feed molar ratios (10, 25 and 50) by also using a catalyst. A PGA sample (59 repetitive units estimated by ^1^H-NMR) with an M_n_ of 13 KDa (value obtained by GPC in DMF) was used [34].

**Figure 1:**
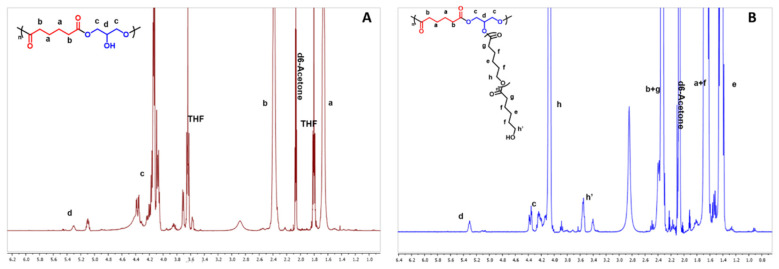
^1^H-NMR spectra of PGA (**A**) and PGA-g-PCL25 (**B**). Only the linear structure of PGA has been reported for sake of clarity: full structure reported in Scheme 1.

Following the published protocol from the Kressler group, the ^1^H-NMR analysis was employed both to confirm the success of the degree of functionalization of PGA, as well as to estimate the length of the CL arms. In the spectrum of the pristine PGA (Figure 1A), the signals of the CH_2_ groups of the adipate unit were at 2.4 to 1.6 ppm, while the glycerol CH was at 4.1 ppm when linked to the free OH group, at 5.1 ppm when linked to CH_2_-OH and at 5.3 ppm when linked to the ester oxygen or to the CH_2_ bonded to the ester oxygen. The end-group methylene groups were at 3.7 ppm, and the CH nearby the end-group at 3.8 ppm. The signals at 3.5 ppm were related to the CH_2_ bonded to the free OH group or to the adipate ester oxygen, which was related to glycerol esterification in the 1,2 position rather than 1,3. The glycerol CH_2_ groups, either in the main chain or as end groups were at 4, 4.2 ppm and 4.9 ppm.

The presence of 1,3-disubstituted (target reaction), 1,2-disubstituted and 1,2,3-trisubstituted glyceride groups (side events) in PGA has been already described, with the latter leading to polymer branching as well as to the decrease in the number of polymer hydroxyl groups [17]. The methine proton from the 1,2,3-trisubstituted glycerol unit at 5.3 ppm was used to calculate the PGA degree of branching, by comparing the integrals of this peak and the CH_2_ peaks of the adipic units at 1.66 and 2.39 ppm, as previously reported [34]. In our polymer, the degree of branching resulted in ca. 5%.

Similarly, the degree of functionalization of PGA with PCL, which was determined by comparing the integral of the peak at 5.3 ppm (d, related to PGA functionalization) and that at ca. 3.6 ppm (h’, related to the end group -CH_2_OH of PCL arms on PGA) resulted in ca. 95%, in agreement with previous works [35].

The length of the PCL chain grafted to PGA was, instead, determined by considering the peaks at 4.1 ppm (h) of PCL and at 3.6 ppm (h’) related to the end group -CH_2_OH of PCL arms on PGA. The number of CL repeating units linked to PGA were 10, 22 and 48 for PGA-g-PCL10, PGA-g-PCL25 and PGA-g-PCL50, respectively (Figure 1B).

### 2.1. Thermal Analysis of the Synthesized Polymers

**Figure 2:**
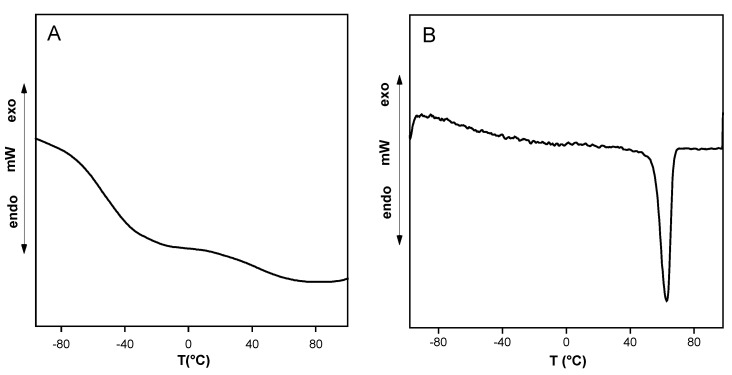
DSC thermograms of the homopolymers PGA (**A**) and PCL10 (**B**).

The thermal properties of the synthesized homopolymers and copolymers were studied by DSC. In Figure 2A,B, the thermograms of PGA and PCL10 are reported as an example.

PGA was essentially an amorphous polymer, with a glass transition (T_g_) at circa −54 °C, while, as expected, PCL had a semicristalline nature with a melting peak centered at circa 60 °C (Figure 2A). In Table 1, the thermal data of all of the synthesized copolymers are reported. As for the PCL homopolymers, a slight increase in the melting temperature (T_m_) of the polymers was observed with the increase in the PCL arm length. This finding suggests an enhancement in the cohesion forces among polymer chains.

**Figure 3:**
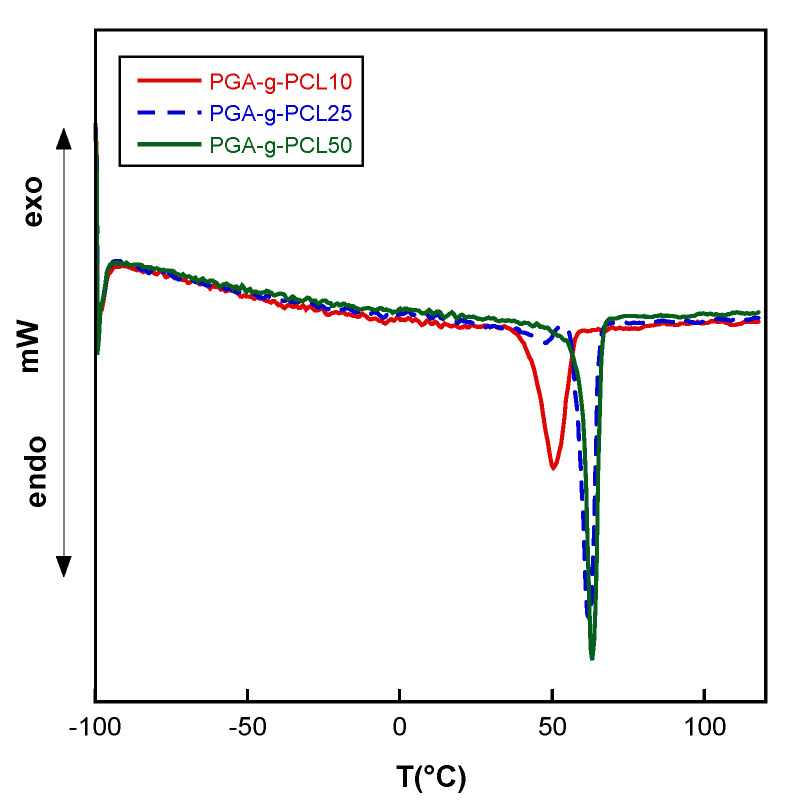
DSC thermograms of PGA-g-PCL copolymers.

As for the PGA-g-PCL copolymers, they were semicristalline polymers (Figure 3) with a melting temperature depending on the length of the PCL side chain. In addition, a decrease in the melting temperature of the PCL block was observed with respect to the PCL homopolymers with the same length (Table 1), presumably suggesting some miscibility between the two polyesters, especially for shorter PCL arm lengths. In contrast, a crystallinity increase in the materials was observed with the increase in the PCL arm length (see ΔH_m_ values in Table 1).

The obtained results in terms of the melting temperatures of the copolymers are consistent with those obtained by Pfefferkon et al. [42], who modified PGA with single and double crystallizable chains (PCL and PEO). In the two synthesized PGA-g-PCL copolymers, PGA17-g-PCL15 and PGA17-g-PCL24, the authors found that PCL was able to crystallize after grafting, retaining a spherulite morphology, even with a lower spherulite radius compared to the PCL homopolymers. In addition, the SAXS patterns evidenced how the PCL chains in PGA17-g-PCL15 adopted an extended chain configuration, whereas in PGA17-g-PCL24, the chains were once-folded, with a slightly smaller lamella thickness compared to the corresponding homopolymers.

### 2.2. Preparation of Usnic Acid-Loaded Nanoparticles

PGA and PGA-g-PCL copolymers were used as carriers for the hydrophobic antimicrobial drug usnic acid. As described in the Section 3, the solvent displacing method was used for the nanoparticle preparation and UA loading. In the adopted conditions, the amount of UA loaded into the NPs was almost complete with an encapsulation efficiency ranging from 95 to 99%. Therefore, drug loading was considered to be 0.3 mg of UA per mg of nanoparticles ($\frac{0.3\;mg\;of\;UA}{mg\;of\;NPs}$), i.e., the UA/nanoparticle ratio used for nanoparticle preparation.

To gain information about the drug fraction physically adsorbed onto the nanoparticle surface with respect to the drug entrapped in the nanoparticle core, NPs were washed repeatedly with methanol: a good solvent for usnic acid. Findings showed that in the case of PGA-g-PCL25 and PGA-g-PCL50, about 95% of the drug was released in methanol, suggesting an adsorption of the drug mainly on the NP surface. In contrast, only 65% of the loaded UA drug was released in the case of PGA-g-PCL10, suggesting a greater UA loading in the NP core. These findings may be justified by considering the higher degree of crystallinity of the polymers with the increasing PCL length [9,10,11].

The size of the obtained polymer nanoparticles before and after drug loading is reported in Table 2.

As can be observed, the introduction of PCL as an arm in the PGA backbone induced a remarkable reduction of the nanoparticle size thanks to the interactions among PCL chains. When UA was loaded into the nanoparticles, a decrease in size was observed for the PGA, while a slight, not significant, increase was found for the PGA-g-PCL copolymers. The decrease in PGA nanoparticle size following UA loading is peculiar and may be related to the establishment of drug/polymer interactions (presumably both hydrophobic and H-bond interactions), which promoted self-assembly of the system. A similar NP size decrease was found for the loading of ibuprofen sodium salt into plain and acylated-PGA nanoparticles. The authors related their findings to the establishment of strong drug–polymer interactions, as confirmed by the DSC analysis performed on ibuprofen-loaded PGA nanoparticles, where a molecular dispersion of the drug into the system was found [44].

### 2.3. Kinetics of Usnic Acid Release from Nanoparticles

**Figure 4:**
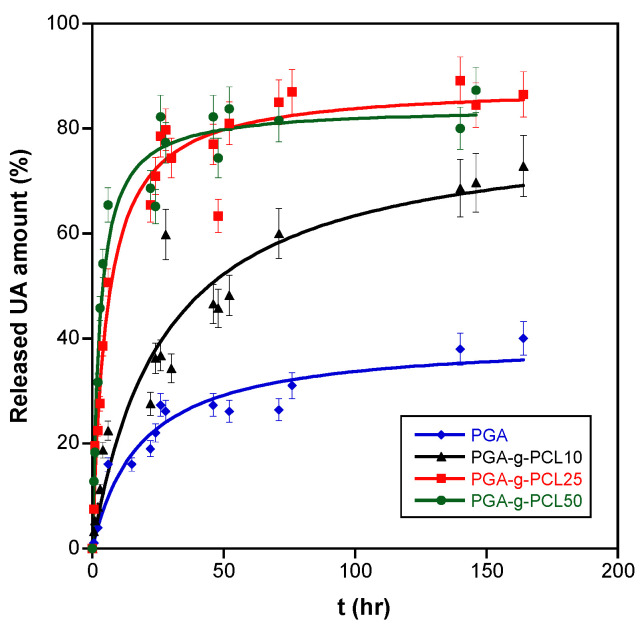
Kinetics of drug release from PGA and PGA-g-PCL nanoparticles in PBS (pH = 7.4) at 25 °C.

The UA release from nanoparticles was studied in PBS (pH = 7.4) at 25 °C. As can be observed in Figure 4, plain PGA nanoparticles released less UA at equilibrium (ca. 35%), supporting the hypothesis of a good drug/polymer interaction presumably related to the establishment of H-bond interactions between UA phenol groups and PGA OH groups, other than hydrophobic interactions. This result is consistent with the observed decrease in size of the UA-loaded PGA nanoparticles compared to empty nanoparticles. 

The introduction of PCL arms in the PGA backbone increased both the total amount of released UA (more than 80%) and the UA release rate. That was accentuated when longer PCL chains were introduced (PCL25 and PCL50), where a burst initial release was observed. Presumably, in case of long PCL arms grafted to PGA, the drug is adsorbed preferentially onto the NP surface. Drug penetration in the NP core may be hindered by PCL hydrophobicity and crystallinity, which make water penetration more difficult.

Nevertheless, all of the systems, especially PGA-g-PCL10, exhibited a prolonged UA release at least up to 7 days (172 h).

In order to investigate the mechanism of drug release, the Korsemeyer–Peppas model was applied. In Table 3, the values of the transport exponent (*n*) are reported for all of the systems. In general, for *n* = 0.45, the release was a diffusion-controlled, Fickian mechanism of release (Case I diffusional), even if *n* may depend on carrier geometry (0.50, 0.45 and 0.43 for release from slabs, cylinders and spheres), and in the case of spherical polymer particles, it depended on the width of the distribution [45]. For 0.45 < *n* < 0.89, drug release was anomalous, and non-Fickian. For *n* = 0.89, we had the Case II transport (polymer relaxation- or swelling-controlled mechanism). For *n* ≥ 0.9, the drug release was controlled by polymer swelling and erosion.

As can be observed, for PGA and PGA-g-PCL10, a Fickian diffusion-controlled release was observed. In contrast, an anomalous release mechanism was evidenced for PGA-g-PCL25 and PGA-g-PCL50, where presumably both diffusion and polymer relaxation contributed to the drug release.

**Figure 5:**
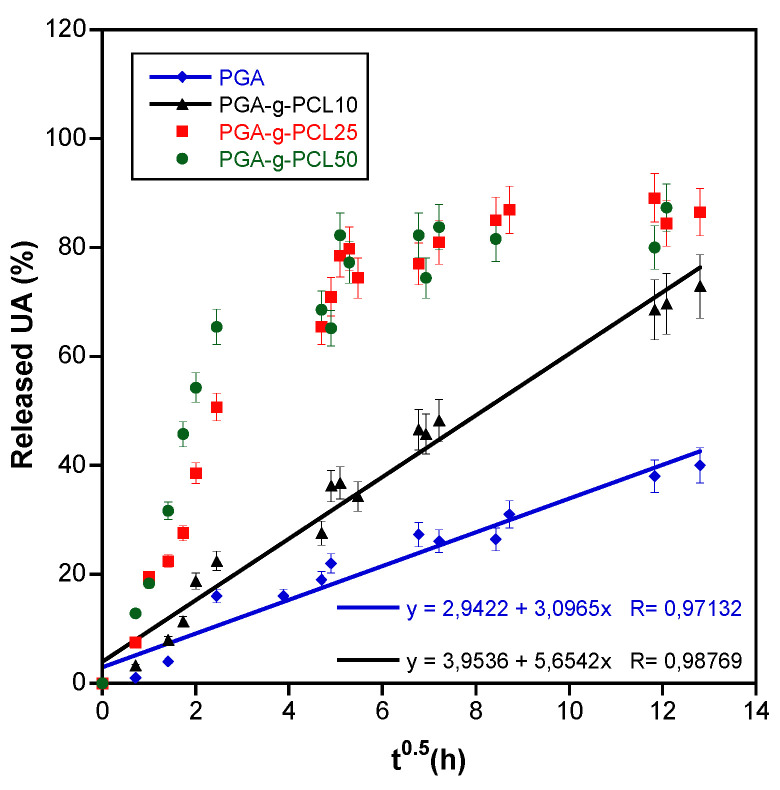
Higushi plot: released UA (%) vs. the root square of time.

The application of the Higushi model confirmed a diffusion-controlled release mechanism for PGA and PGA-g-PCL10 as can be observed by the good linear regression of the release data vs. the root square of time (Figure 5). No linear fitting was found for the PGA-g-PCL25 and PGA-g-PCL50 release data.

The Higushi dissolution constant (K_H_), related to the drug diffusion coefficient in the system and determined from the slope of the linear regression, was 5.6 for PGA-g-PCL10 and 3.1 for PGA. The higher K_H_ found for PGA-g-PCL10 compared to PGA suggested a favored diffusion of the drug in the presence of PCL arms, which were presumably disturbing PGA self-assembling.

The anomalous release mechanism observed for PGA-g-PCL25 and PGA-g-PCL50 may suggest that these systems were substantially not swellable due to the hydrophobicity and crystallinity of long PCL arms [9,10,11]. Indeed, the non-Fickian anomalous release was first described by Ritger and Peppas for non-swellable cylinders and spherical samples [45].

### 2.4. Antimicrobial Activity of UA-Loaded Nanoparticles

**Figure 6:**
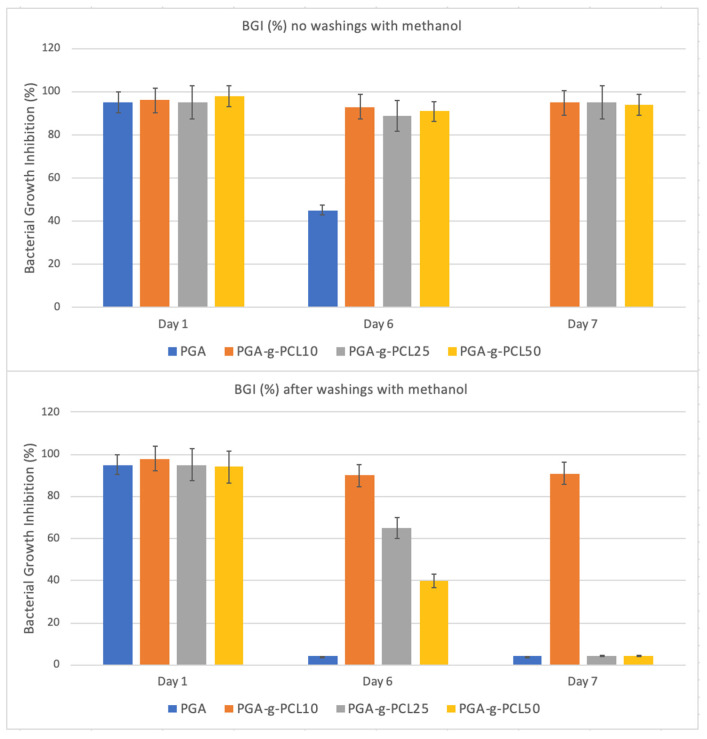
Bacterial growth inhibition percentage (BGI, %) of the UA-loaded nanoparticles before and after washings with methanol. Strain: *Staphylococcus epidermidis*.

All of the UA-loaded NP systems showed a significant and prolonged antimicrobial activity towards a standard strain of *Staphylococcus epidermidis*. Specifically, all of the PGA-g-PCL copolymers were able to cause a complete bacterial growth inhibition, determined as described in Section 3, up to at least 7 days of the bacterial challenge (Figure 6).

This finding was related to the high UA loading (0.3 mg/mg of NP) achieved for all of the systems, which permitted the release of efficacious drug amounts at least up to 7 days.

To highlight the differences among the samples, the antimicrobial test was performed on the nanoparticle systems after being repeatedly washed with methanol (a good solvent for UA), which eliminated the weakly adsorbed drug. The durability of the activity of the systems was found to be different and related to the amount of drug released in methanol. Specifically, only PGA-g-PCL10, which released the lowest drug amount in methanol (65% vs. 95% of the other systems) showed a long-lasting activity (up to 7 days) also after the washings in methanol.

Usnic acid has great therapeutic potential, but its use in clinics is largely hindered by its poor solubility in water, and availability. This explains why the development of nanocarriers for usnic acid is under investigation by a number of research groups, with variable success mainly depending on the nature of the investigated carrier. Rauschenbach et al. [26] recently reported the complexation of usnic acid with amphiphilic, stimuli-responsive, biodegradable, hyperbranched polymers showing powerful antimicrobial activity against the Gram-positive *Staphylococcus aureus* species. However, no information was provided about the durability of the activity. Similarly, an increase in the activity of usnic acid against *Mycobacterium tuberculosis* as well as cellular uptake into macrophages were reported after UA encapsulation into liposomes [28]; however, also in this case, the duration of the antimicrobial activity was not investigated. To the best of our knowledge, this is the first investigation in which the activity of UA-loaded nanosystems was investigated over time. In general, to prolong the antimicrobial activity of polymer systems, several strategies have been investigated, including the incorporation of drugs or drug-loaded nanoparticles in polymer matrices with or without pore former agents [46,47]. In our case, the macromolecular architectural changes produced by PCL grafted from PGA were able to significantly influence the drug release profile and mechanism. A long-lasting antimicrobial activity is relevant for several clinical applications including the implantation of medical devices, where the risk of infection is definitely high in the first week following implant insertion, and the treatment of chronic wounds, where the availability of a long-lasting releasing nanosystem would permit the prevention/treatment of bacterial infections, thus reducing the number of interventions on the wound.

Overall, the developed nanocarriers seem to be promising tools for the administration of usnic acid or different therapeutic agents with physico–chemical features similar to usnic acid.

## 3. Materials and Methods

### 3.1. Materials

Caprolactone (CL, Sigma Aldrich, ST. Louis, MO, USA) was distilled before use. Polyglyerol adipate (PGA) was synthesized and purified as previously reported [34]. Tetrahydrofuran (THF, Sigma Aldrich) was dehydrated on CaSO_4_. Tin 2-ethylexanoate (Sigma Aldrich), methanol (Sigma Aldrich), hexane (Sigma Aldrich) and usnic acid (Sigma Aldrich) were used without further purification.

### 3.2. Polymer Synthesis

**Scheme 1:**
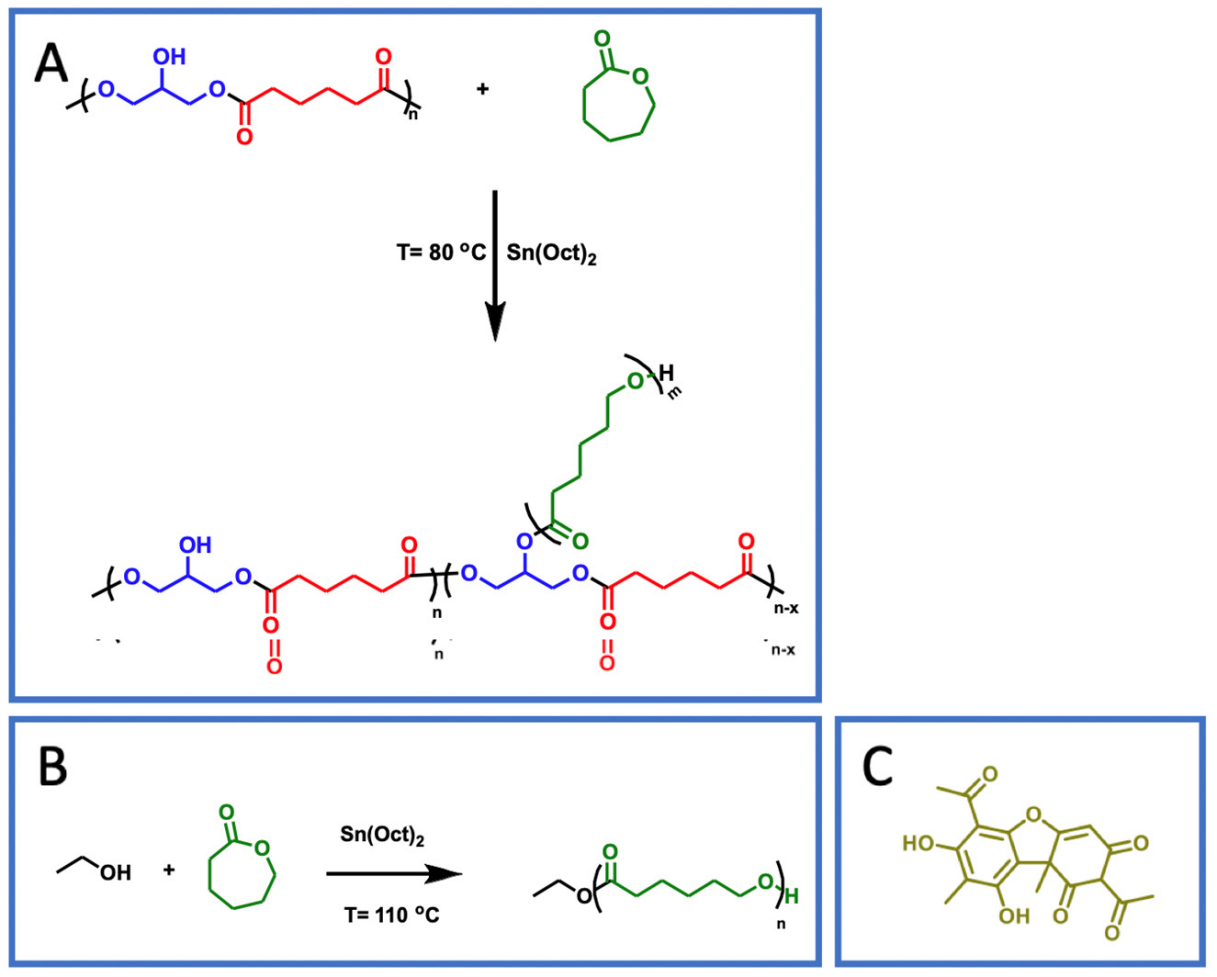
Chemical structures of PGA-g-PCLs (**A**) and of PCL homopolymer (**B**) and usnic acid (**C**).

The synthesis of the PCL-grafted PGAs was carried out following the procedure adopted by Pfefferkonet et al. [42]. In particular, distilled CL was added to PGA in tetrahydrofuran at various CL/OH-PGA molar ratios (10, 25 and 50). Then, the catalyst, tin 2-ethylexanoate, was added and the reaction was carried out at 80 °C for 20 h, under stirring. The copolymers were precipitated twice in cold methanol and recovered by centrifugation. After two washings in hexane to eliminate the catalyst, the materials were dried in a vacuum oven at 40 °C for 1 day. The obtained copolymers were named PGA-g-PCL10, PGA-g-PCL25 and PGA-g-PCL50. In Scheme 1, the chemical structure of copolymers is reported.

PCL homopolymers, with chain lengths similar to those in the PGA-g-PCL copolymers, were also synthesized by using ethanol as an initiator for the ring opening polymerization of caprolactone (Scheme 1). Specifically, CL and ethanol were mixed in proper ratios at room temperature and under nitrogen flow. Then, the temperature was raised to 110 °C, and the catalyst tin 2-ethylexanoate was added. The reaction was left under stirring for 24 h. After the reaction, THF was added at room temperature to solubilize the polymer, which was then precipitated in cold methanol. Finally, polymer samples were dried in the vacuum oven at 40 °C for 1 day. The obtained polymers were named PCL10, PCL25 and PCL50.

### 3.3. Polymer Characterization

A proton nuclear magnetic resonance (^1^H-NMR) analysis was carried out by a VARIAN XL 300 spectrometer, by using tetramethylsilane as the internal reference. Samples were dissolved in deuterated acetone ((CD_3_)_2_CO), 2.04 ppm).

Differential scanning calorimetry (DSC) was performed by a Mettler TA-3000 DSC apparatus. Thermograms were acquired at 10 °C min*^−^*^1^ in the −100 ÷ 120 °C temperature range, under N_2_ flux. 

### 3.4. Preparation and Characterization of Polymer Nanoparticles

Polymer nanoparticles, alone or containing usnic acid, were prepared by the solvent displacement method.

Polymers (60 mg) were dissolved in acetone (10 mL) and added dropwise to water (15 mL) under magnetic stirring. The solution was then left under stirring for 24 h at room temperature in order to evaporate the acetone. The precipitated particles were finally recovered by centrifugation (3500 rpm for 5 min) and freeze-dried.

Usnic acid-entrapped nanoparticles were prepared with the same experimental conditions described above, with the only difference being that polymers (60 mg) were dissolved in acetone (10 mL) containing 18 mg of usnic acid. Furthermore, in this case, the precipitated particles were recovered by centrifugation and freeze-dried.

The amount of the loaded drug was evaluated by UV-vis spectroscopy at 280 nm by determining the amount of the drug remaining in the supernatant after centrifugation. The encapsulation efficiency (*EE*) was defined as the ratio of the actual and original amount of usnic acid encapsulated in the nanoparticles and determined with the following equation:

$$EE=\frac{\mathrm{Actual}\;\mathrm{drug}\;\mathrm{amount}\;\mathrm{loaded}\;\mathrm{in}\;NPs}{\mathrm{Theoretical}\;\mathrm{drug}\;\mathrm{amount}\;\mathrm{loaded}\;\mathrm{in}\;NPs}\times 100$$

To have an estimation of the amount of the drug physically adsorbed onto the nanoparticle surface with respect to the drug entrapped in the nanoparticle core, nanoparticles were washed repeatedly with methanol, i.e., a good solvent for usnic acid. The amount of the drug present in methanol was determined by UV-vis spectroscopy at 280 nm.

The size of the nanoparticles was determined by a Laser Diffraction Particle Size Analyzer, LS 13320, Beckman Coulter (Pasadena, CA, USA), by using water as the suspending medium.

### 3.5. Drug Release

The release study was carried out in a phosphate buffer solution (PBS), pH 7.4, at room temperature. Polymer nanoparticles were inserted in a dialysis tube (CUTOFF 3500 Da) and immersed in PBS. At determined times, a solution aliquot was withdrawn and analyzed by UV-vis spectroscopy at 280 nm to determine the amount of the drug in the solution. Each experiment was conducted in triplicate. After the analysis, the withdrawn medium was put back in the glass tube to continue the release experiments. The cumulative release fraction was expressed as *M*_(*t*)_/*M*_0_, where *M*_0_ was the amount of the drug in the microspheres at the beginning, and *M*_(*t*)_ was the drug released at the time *t*.

To investigate the mechanism of the drug release, the experimental data were elaborated by using two models: (i) the Korsemeyer–Peppas model [48] and (ii) the Higushi model, applicable to study the release of water-soluble and low-soluble drugs incorporated in solid matrices [49].

In the Korsemeyer–Peppas equation:

$$\frac{M_{t}}{M_{\infty }}=Kt^{n}$$

*M_t_/M_∞_* represents the fractional released drug, t is the time, *K* is the release constant and *n* is the transport exponent (dimensionless). *K* provides mostly the information on structural features of the nanocarriers, whereas n is related to the drug release mechanism (Fickian diffusion or non-Fickian diffusion). This model also shows the release behavior only for the first 60% of the release of the drug ($\frac{M_{t}}{M_{\infty }}\leq 60\%$) [50].

The Higushi model can be applied when the release is governed by the diffusion (Fick law J = −D ∂C/∂x), and involves that the drug release is a process dependent on the square root of time:

$$Q_{t}=(2D\;Cs\;{\left(A-0.5Cs\right)}^{0.5})\;t^{0.5}\;or\;Q_{t}=K_{H}t^{0.5}$$

where *D* is the diffusion coefficient, *Cs* is the drug solubility, *A* is the drug content for formulation unit and *K_H_* is the Higushi dissolution constant.

### 3.6. Antimicrobial Activity

A reference strain of *S. epidermidis* (ATCC 35984) was employed in the experiments. A determined nanoparticle amount (2.5 mg) was immersed in 2.5 mL of a bacterial suspension (10^8^ CFUs/mL, 0.125 OD at 550 nm) in Muller Hinton (MH) broth and incubated for 24 h at 37 °C. Following incubation, nanoparticles were separated by centrifugation and the bacterial culture was harvested. The antibacterial effect was assessed by measuring the absorbance of the broth at 550 nm. At each tested concentration, the bacterial growth inhibition (*BGI*) was determined as follows:

$$BGI=1-\left(\frac{A_{test\;tube}-A_{0}}{A_{control}-A_{0}}\right)\times 100$$

where *A_test tube_* and *A_control_* are the absorbances of the test and the control tube after 24 h incubation, and *A*_0_ is the absorbance of the bacterial suspension before incubation. Tubes without nanoparticles served as controls.

To evaluate the durability of the antimicrobial activity of the nanoparticles, the test was repeated daily by transferring the same nanoparticles into a new test tube containing a freshly prepared bacterial inoculum.

### 3.7. Statistics

One-way analyses of variance comparisons were performed using MiniTab. Differences were considered significant for *p* values < 0.05. Data were reported as means ± standard deviation.

## 4. Conclusions

Over the last two decades, several nanoparticle antibiotic delivery systems have been developed, and some of them have also reached the clinics. Nevertheless, a real concern related to the use of these nanosystems is the potential reduction of their therapeutic efficacy due to the loss of activity of conventional antibiotics as a consequence of the selection of multi-drug-resistant microorganisms. In this framework, the use of antimicrobial agents not commonly used in clinics is highly recommended for the development of antimicrobial nanosystems. Usnic acid is a secondary metabolite of lichens with great antimicrobial activity not conventionally used for infection treatment because of its poor water solubility and hepatotoxicity.

In the present work, the first in vitro characterization of a polyglycerol adipate-based nanoparticle delivery system for usnic acid was carried out. PGA was enzymatically synthesized under mild conditions and a renewable hydrophilic building block such as glycerol was used as a replacement for the PEGylated chains. Thanks to the nature of its monomers and reaction conditions, PGA resulted in a biodegradable and biocompatible polyester able to spontaneously self-assemble in water into NPs. Our findings demonstrated the potential of the developed systems to ensure prolonged and strong bactericidal activity against a model bacterial species, *S. epidermidis*. PCL grafting to PGA did not affect drug loading but significantly influenced the drug release profile and mechanism. By varying the length of PCL arms, it was possible to tune the drug release from a burst anomalous drug release (high PCL chain length) to a slow diffusion-controlled release (low PCL chain length). That resulted in a prolonged antimicrobial activity of the developed systems, which could be used in preventing/treating infections occurring at different body sites, including mucosal/skin surfaces where Gram-positive bacteria are commonly involved.

## Figures and Tables

**Table 1:** Melting temperature (T_m_) and melting enthalpy (**Δ**H) of the PCL homopolymers and PGA-g-PCL copolymers.

Polymer	T_m_ (°C)	ΔH (J/g)
PCL10	62	98
PCL25	65	100
PCL50	66	90
PGA-g-PCL10	54	66
PGA-g-PCL25	61	79
PGA-g-PCL50	62	88

**Table 2:** Size of PGA and PGA-g-PCL nanoparticles with or without usnic acid.

Polymer	Size (nm) without UA	Size (nm) Loaded with UA
PGA	180 ± 20	110 ± 10
PGA-g-PCL10	90 ± 4	108 ± 9
PGA-g-PCL25	95 ± 3	102 ± 8
PGA-g-PCL50	89 ± 4	109 ± 8

**Table 3:** Korsemeyer–Peppas transport exponent (*n*) and correlation coefficient (R) for the UA-loaded nanoparticles.

Polymer	*n*	R
PGA	0.37 ± 0.06	0.97
PGA-g-PCL10	0.38 ± 0.04	0.97
PGA-g-PCL25	0.68 ± 0.04	0.99
PGA-g-PCL50	0.75 ± 0.03	0.99

## Data Availability

Not applicable.
